# Genetic insight into the putative causal proteins and druggable targets of osteoporosis: a large-scale proteome-wide mendelian randomization study

**DOI:** 10.3389/fgene.2023.1161817

**Published:** 2023-06-28

**Authors:** Zhichong Wu, Kenneth Guangpu Yang, Tsz-Ping Lam, Jack Chun Yiu Cheng, Zezhang Zhu, Wayne Yuk-Wai Lee

**Affiliations:** ^1^Division of Spine Surgery, Department of Orthopedic Surgery, Nanjing Drum Tower Hospital, The Affiliated Hospital of Nanjing University Medical School, Nanjing, China; ^2^Musculoskeletal Research Laboratory, SH Ho Scoliosis Research Laboratory, Department of Orthopaedics and Traumatology, Faculty of Medicine, The Chinese University of Hong Kong, Shatin, Hong Kong SAR, China; ^3^ Joint Scoliosis Research Centre of the Chinese University of Hong Kong and Nanjing University, The Chinese University of Hong Kong, Shatin, Hong Kong SAR, China; ^4^ Prince of Wales Hospital, Li Ka Shing Institute of Health Sciences, The Chinese University of Hong Kong, Shatin, Hong Kong SAR, China; ^5^ Center for Neuromusculoskeletal Restorative Medicine, CUHK InnoHK Centres, Shatin, Hong Kong SAR, China; ^6^Key Laboratory for Regenerative Medicine, School of Biomedical Sciences, Faculty of Medicine, Ministry of Education, The Chinese University of Hong Kong, Shatin, Hong Kong SAR, China

**Keywords:** mendelian randomization, osteoporosis, BMD, genetic, drug

## Abstract

**Background:** Osteoporosis is a major causative factor of the global burden of disease and disability, characterized by low bone mineral density (BMD) and high risks of fracture. We aimed to identify putative causal proteins and druggable targets of osteoporosis.

**Methods:** This study utilized the largest GWAS summary statistics on plasma proteins and estimated heel BMD (eBMD) to identify causal proteins of osteoporosis by mendelian randomization (MR) analysis. Different GWAS datasets were used to validate the results. Multiple sensitivity analyses were conducted to evaluate the robustness of primary MR findings. We have also performed an enrichment analysis for the identified causal proteins and evaluated their druggability.

**Results:** After Bonferroni correction, 67 proteins were identified to be causally associated with estimated BMD (eBMD) (*p* < 4 × 10^−5^). We further replicated 38 of the 67 proteins to be associated with total body BMD, lumbar spine BMD, femoral neck BMD as well as fractures, such as RSPO3, IDUA, SMOC2, and LRP4. The findings were supported by sensitivity analyses. Enrichment analysis identified multiple Gene Ontology items, including collagen-containing extracellular matrix (GO:0062023, *p* = 1.6 × 10^−10^), collagen binding (GO:0005518, *p* = 8.6 × 10^−5^), and extracellular matrix structural constituent (GO:0005201, *p* = 2.7 × 10^−5^).

**Conclusion:** The study identified novel putative causal proteins for osteoporosis which may serve as potential early screening biomarkers and druggable targets. Furthermore, the role of plasma proteins involved in collagen binding and extracellular matrix in the development of osteoporosis was highlighted. Further studies are warranted to validate our findings and investigate the underlying mechanism.

## Introduction

Characterized by low bone mineral density (BMD) and impaired bone microarchitecture, osteoporosis is the most common skeletal disorder (Compston et al., 2019). The prevalence of osteoporosis worldwide was reported to be 18.3%, and the number keeps increasing rapidly due to the aging of the population (Salari et al., 2021). Low BMD and osteoporosis may lead to the fragility of bone and increased risks of fracture, resulting in substantially higher healthcare burden, risks of disability, and mortality (Cummings and Melton, 2002; Shen et al., 2022). The typical treatment regimen for osteoporosis encompasses pharmaceutical interventions, including bisphosphonates and denosumab. However, these agents have the potential to elicit adverse effects, including gastrointestinal disturbance, musculoskeletal pain, and the rare complication of osteonecrosis of the jaw. Emerging evidence on molecular drug targets provide new viewpoint to treatment of OP.

Proteins are the major source of druggable targets and serve as biomarkers of complex traits (Santos et al., 2017; Storm et al., 2021). Previous observational studies have established correlations between protein levels and BMD (Martínez-Aguilar et al., 2019; Huang et al., 2020; Al-Ansari et al., 2022), but the findings may be biased by confounders and reverse effects, leaving the causal relationship remains unclear. Mendelian randomization (MR), similar to randomized control trials (RCT), is a novel statistical approach that uses genetic variants (i.e., protein quantitative trait loci, pQTLs) as proxies to infer the causality between exposures (i.e., proteins) and outcomes (i.e., BMD) (Sanderson et al., 2022). Dissecting causal relationships not only lead to the identification of the driving proteins of human disease but also provide strong evidence of druggable targets. Due to the limited sample size and lack of statistical power of GWAS on proteins, Previous MR studies focused more on the effect of gene methylation and gene expression on complex traits (Liu et al., 2021a; Liu et al., 2021b; Chen et al., 2022), leaving the direct effect of proteins being largely uncharacterized. With significant technological advances in proteomic profiling and a substantial reduction in the cost of genomic sequencing, genetic determinants of over 4,000 proteins have recently been reported by large-scale GWAS studies of plasma protein which offers us a valuable opportunity to comprehensively address the causal relationship between proteins and multiple diseases (Suhre et al., 2017; Sun et al., 2018; Ferkingstad et al., 2021). Through the integration of genomic and proteomic data by MR, a few studies have successfully identified druggable targets and biological pathways of human disease, such as childhood neurodevelopmental disorders (Yang et al., 2022), COVID-19 (Palmos et al., 2022; Zheng et al., 2022) and depression (Deng et al., 2022). However, genomic insights into the causal proteins, as well as druggable target of osteoporosis have not been comprehensively explored.

In this study, we aimed to prioritize causal proteins and druggable targets of osteoporosis using large-scale proteome-wide mendelian randomization by combining the largest publicly available GWAS datasets on plasma proteins and osteoporosis-related traits.

## Methods

### Source of exposure and outcome datasets

Exposures for MR were plasma-circulating proteins, and outcomes were bone mineral density and fractures (Table 1). The largest and most comprehensive GWAS summary datasets on plasma protein were obtained from the consortium of deCODE genetics (https://www.decode.com/summarydata/) (Ferkingstad et al., 2021). The detailed description of the datasets can be found in the original report. In brief, the pQTL datasets consisted of the associations between genome-wide genetic variants and 4,719 plasma proteins tagged by 4,907 aptamers in 35,559 Icelanders adjusted for age, sex, and sample age. The plasma protein levels were measured with the SomaScan version 4 assay (SomaLogic).

**TABLE 1:** Source and descriptive Information of GWAS data used in mendelian randomization.

Phenotypes	Ancestry	Sample size	Method of measurement	Adjustments	PMID
Plasma proteins	European	35,559	SomaScan	Age, sex and sample age	34857953
eBMD (Primary analysis)	European	426,824	Ultrasound	Age, sex, genotyping array, assessment center, and ancestry informative principal components 1 to 20	30598549
TBBMD (Secondary analysis)	European	56,284	DXA	Age, sex, weight, height, genomic principal components, study-specific covariates	29304378
LSBMD (Secondary analysis)	Predominantly European (∼70%)	44,731	DXA	Sex, age, age^2^, weight	26367794
FNBMD (Secondary analysis)	Predominantly European (∼70%)	49,988	DXA	Sex, age, age^2^, weight	26367794
Fracture (Secondary analysis)	European	426,795 (53,184 cases)	Hospital-based and self-reported fracture history	Age, sex, genotyping array, assessment center, and ancestry informative principal components 1 to 20	30598549

In the discovery phase for the causal proteins of osteoporosis, we utilized the largest GWAS datasets on BMD estimated from quantitative heel ultrasounds (eBMD), which includes 426,824 individuals (Morris et al., 2019). Secondary MR analysis was performed to validate the identified proteins on other osteoporosis-related traits, including total bone BMD (TBBMD), femoral neck BMD(FNBMD), lumbar spine BMD (LSBMD), and fractures. The datasets of eBMD and fracture were obtained from UK Biobank (https://www.ukbiobank.ac.uk/) which is a large-scale biomedical database and research resource, containing genetic and phenotypic information from nearly 500,000 participants. A total of 426,795 white British individuals, comprising 53,184 cases and 37,361 controls, were included in the fracture GWAS (Morris et al., 2019). The identification of fracture cases was based on hospital-based fracture diagnosis and self-reported fracture within the past 5 years. The summary-level data on dual-energy X-ray absorptiometry (DXA) derived TBBMD (*n* = 56,284) (Medina-Gomez et al., 2018), FNBMD (*n* = 49,988) and LSBMD (*n* = 44,731) (Zheng et al., 2015a) were accessed from GEnetic Factors for OSteoporosis Consortium (GEFOS) (http://www.gefos.org/).

To the best of our knowledge, most participants were of European ancestry and there is no sample overlap between exposure and outcome datasets which minimizes the bias from population stratification and overlapped participants.

### Selection of instrumental variables

The selection of instrumental variables (IVs) were based on three assumptions (Sanderson et al., 2022). First, the IVs should be associated with exposures (proteins). Second, the IVs are supposed to be independent of confounders. Third, the effects of IVs on outcomes were only mediated through exposures.

To satisfy the first assumption, we selected genetic variants that were associated with corresponding proteins using a stringent *p*-value threshold (*p* < 5 × 10^−8^). To avoid weak instrument bias, we excluded those SNPs with weak strength (F statistic < 10). To meet the second and third assumptions, we removed pleiotropic SNPs [associated with more than five proteins as suggested by previous studies (Zheng et al., 2020; Yang et al., 2023)] and only included SNPs that explain substantially larger variance of exposure than outcomes as indicated by the Steiger filtering test (*p* < 0.05). Given the complex linkage disequilibrium (LD) structure in the region of human major histocompatibility complex (MHC) (chr6:28477897-33448354), the SNPs within the region were removed. To ensure the imputation quality, only genetic variants with minor allele frequency >1% were included (Zheng et al., 2015b). After harmonizing exposure and outcome datasets, we performed linkage disequilibrium (LD) clumping using 1,000 genomes European reference panel to select conditionally independent genetic instruments (r2 < 0.1) (Auton et al., 2015).

There are two types of pQTL according to their genomic location, namely, cis-pQTL (SNPs within 1MB from gene start position) and trans-pQTL (SNPs more than 1MB away from gene start position) (Zheng et al., 2020; Wingo et al., 2021; Ghanbari et al., 2022; Yazdanpanah et al., 2022). The cis-pQTLs were defined as variants located near or within the corresponding protein-coding gene, while trans-pQTLs were distant from the corresponding gene. Comparing with trans-pQTL, cis-pQTLs are more likely to directly affect the genes and are less likely to be prone to horizontal pleiotropy. Therefore, only cis-pQTL within 1MB from gene start position were included in this study.

### Two sample mendelian randomization

To ensure the reliability of MR results, minimize chance finding induced by false positive SNP-protein associations and enable further sensitivity analyses which require at least three instrumental variables (IVs), only proteins with ≥3 instrumental variables (IVs) were included in the following MR analysis.

For the main analysis, MR estimates of each SNP was evaluated by Wald ratios, which were subsequently meta-analyzed using Inverse Variance Weighted (IVW) method. Random-effect IVW model will be used to account for the potential heterogeneity. Furthermore, MR-Egger regression and weighted median model were adopted as sensitivity analyses to evaluate the robustness of MR results. MR-Egger regression combines Wald ratio together into a meta-regression while adjusted for any directional pleiotropy. Generally, MR-Egger regression is more robust to potential pleiotropy, but the statistical power is much lower when compared with IVW method (Burgess and Thompson, 2017). Weighted median model can give a consistent estimate of exposures on outcomes even if up to 50% if IVs were invalid (Bowden et al., 2016). The MR-Egger intercept was conducted to assess the directional horizontal pleiotropy. Cochrane’s Q value was used to assess the heterogeneity. Steiger directionality test was used to evaluate the causal direction between circulating proteins and BMD or fracture.

MR analysis was conducted by TwoSampleMR R package (https://mrcieu.github.io/TwoSampleMR/). The results reaching the threshold of *p*-value < 4 × 10^−5^ (Bonferroni-corrected for 1,245 proteins) were defined as significant in the discovery analysis, by which we identified a list of putative causal proteins.

### Replications of the identified proteins

The causal effects of all the plasma-circulating proteins which reached significance in the primary analysis using MR-IVW method were further replicated on other osteoporosis-related traits, including TBBMD, LSBMD, FNBMD and fractures. TBBMD, LSBMD, FNBMD were measured by DXA which is different from the method of measurement for eBMD, the corresponding population is also independent of the eBMD cohort, and the sample size (*n* = 44,751–56,284) is much smaller than eBMD cohort. The MR methods used in replication phase were identical to those used in the discovery cohort. For the replication and MR-sensitivity analyses, the nominal *p*-value < 0.05 was considered significant.

### GO enrichment analysis and network analysis

To further investigate the biological role of the identified proteins as well as their interactions, we performed Gene Ontology (GO) enrichment analysis and Protein-protein interaction (PPI) network analysis based on the corresponding coding genes. GO enrichment analysis was conducted using enrichGO() function in clusterProfiler package with default settings and the results were adjusted by Benjamini-Hochberg (BH) method. PPI network analysis was conducted using search tool for the retrieval of interacting genes/proteins (STRING) database with default settings (https://string-db.org/). By default, only interactions with medium confidence (>0.4) are presented.

### Druggable genome and known drugs

The druggable genome be defined as a set of protein-coding genes that can or theoretically can be modulated by therapeutic compounds. The products of druggable genes either were already targeted by existing proteins and drugs or have structural and functional properties suggestive of druggability. We obtained the list of druggable genes from the study by Finan et al. (2017) which classified the genes into three tiers according to their druggability. Tier 1 includes the targets of drugs in clinical use or clinical development. Genes in tier 2 have not already be targeted by existing drugs but encode peptides with high sequence homology to tier 1 genes. Tier 3 genes encodes extracellular proteins and members of key drug target families. We further searched for updated information on the drugs targeting the identified putative causal proteins in Open Target platform (https://www.opentargets.org/). The Open Targets Platform is a comprehensive tool that promote identification or drug targets by integrating multiple database.

## Results

### Primary MR analysis identified 67 putative causal proteins on BMD

**FIGURE 1:**
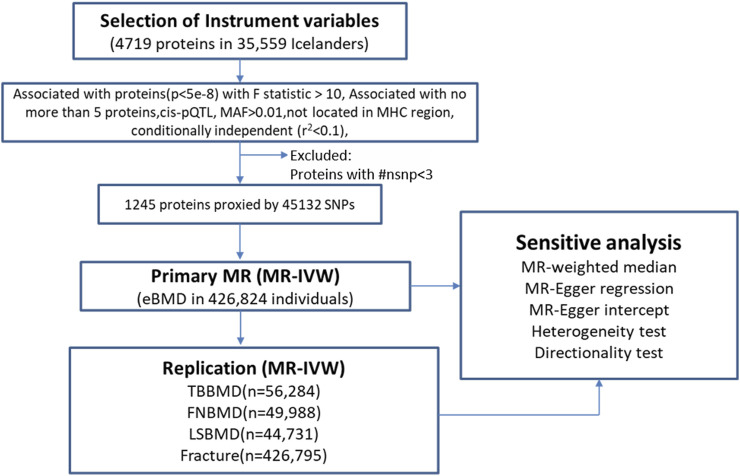
The overall study design and analysis process. MR, mendelian randomization; ##nsnp number of SNPs (IVs); MAF minor allele frequency; MHC, major histocompatibility complex; MR-IVW, mendelian randomization-Inverse Variance Weighted.

**FIGURE 2:**
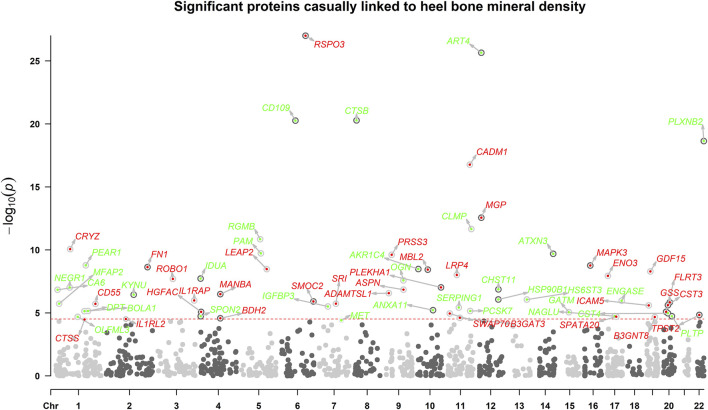
The manhattan plot illustrates the associations between circulating proteins. *X*-axis indicates the position in the chromosome, *y*-axis indicates negative log10-transformed *p*-values. Horizontal dotted line indicates the threshold of Bonferroni-adjusted significance. Red points indicate that the proteins were positively associated with eBMD, while green points indicate negative associations.

The overview of the study design was presented in Figure 1. Based on the up-to-date largest and the most comprehensive GWAS studies, we conducted a two-sample MR analysis to discover putative causal proteins regulating bone mineral density. Following the criteria for the selection of instrumental variables (IVs), there are enough IVs (*n* ≥ 3) for 1,245 aptamers representing 1,215 unique proteins to perform MR analysis, with the number of IVs ranging from 3 to 242 (Supplementary Tables S1, S2). The F-statistic for all the IVs were larger than 10 (Supplementary Table S1), supporting those cis-pQTLs were strong instruments. After corrected for multiple tests by Bonferroni method (*p* < 0.05/1245 or 4 × 10^−5^), our primary MR analysis by IVW method revealed 67 unique proteins, represented by 72 aptamers, to be associated with estimated heel bone mineral density (eBMD) (Figure 2; Supplementary Table S2). Among the 67 proteins, 34 proteins have positive effects on eBMD while the remain 33 proteins were negatively associated with eBMD.

As mendelian randomization may be subjected to pleiotropy of the IVs, several sensitivity analyses were adopted. The effects on eBMD were in the same direction and the estimates were similar for all the identified proteins between IVW MR, weighted median MR and MR-egger regression (Supplementary Table S2). To be noted, all the identified proteins remained to be significant in weighted median MR. Despite low power to detect causal effects, MR-egger method revealed 45 out of the 67 proteins remained significant. Cochrane’s Q test showed low to moderate heterogeneity for the associations between proteins and eBMD. Little evidence of directional horizontal pleiotropy (*p* > 0.05) was found by MR-egger intercept analyses for all the proteins except ART4, CTSB, PRSS3, GDF15, HSP90B1, CST4, TPST2, and NAGLU. Steiger directionality test indicated that MR estimate of causal direction was accurate (All *p* < 0.05).

### 38 proteins replicated to be associated with other osteoporosis-related traits

**FIGURE 3:**
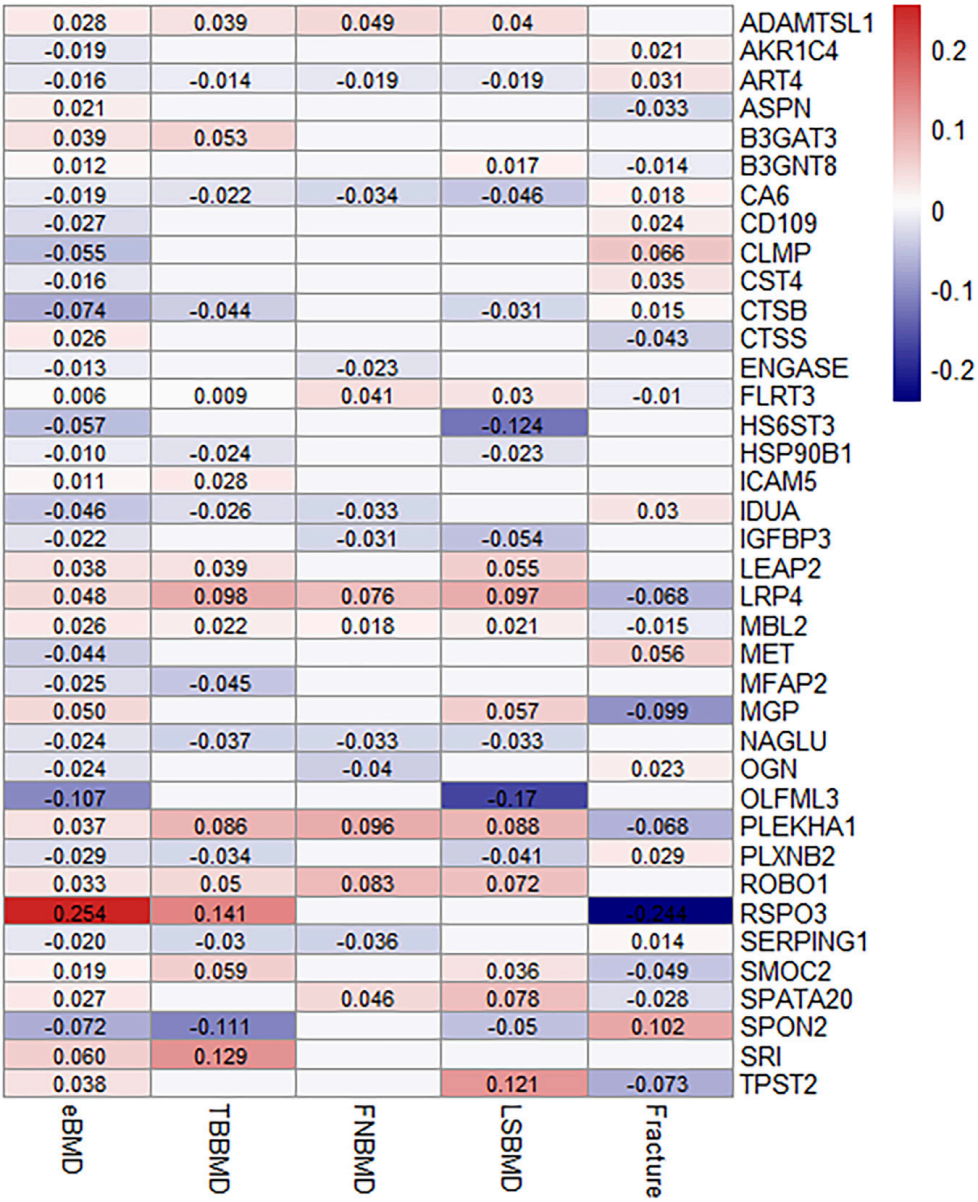
MR-IVW effect estimates (beta value) of proteins that were identified and replicated on osteoporosis-related traits. For aesthetic purposes, the beta values for the insignificant associations were set as zero and not displayed.

Out of 67 identified proteins, 38 proteins were validated to be significantly associated with TBBMD, FNBMD, LSBMD and/or fracture (*p* < 0.05) and in the same direction for all the significant associations, including ADAMTSL1, AKR1C4, ART4, ASPN, B3GAT3, B3GNT8, CA6, CD109, CLMP, CST4, CTSB, CTSS, ENGASE, FLRT3, HS6ST3, HSP90B1, ICAM5, IDUA, IGFBP3, LRP4, MBL2, MET, MFAP2, MGP, NAGLU, OGN, OLFML3, PLEKHA1, PLXNB2, ROBO1, RSPO3, SERPING1, SMOC2, SPATA20, SPON2, SRI, and TPST2 (Figure 3; Supplementary Table S3). The results were largely consistent in multiple sensitivity analyses (Figure 3; Supplementary Table S3). Furthermore, the genetically predicted causal effects of the 67 identified proteins on BMD at different anatomical sites demonstrate positive correlations (*r* = 0.33–0.65), and exhibit negative associations with fracture risk (*r* = −0.72–−0.33) (Supplementary Table S4).

### GO enrichment analysis and PPI networks

**FIGURE 4:**
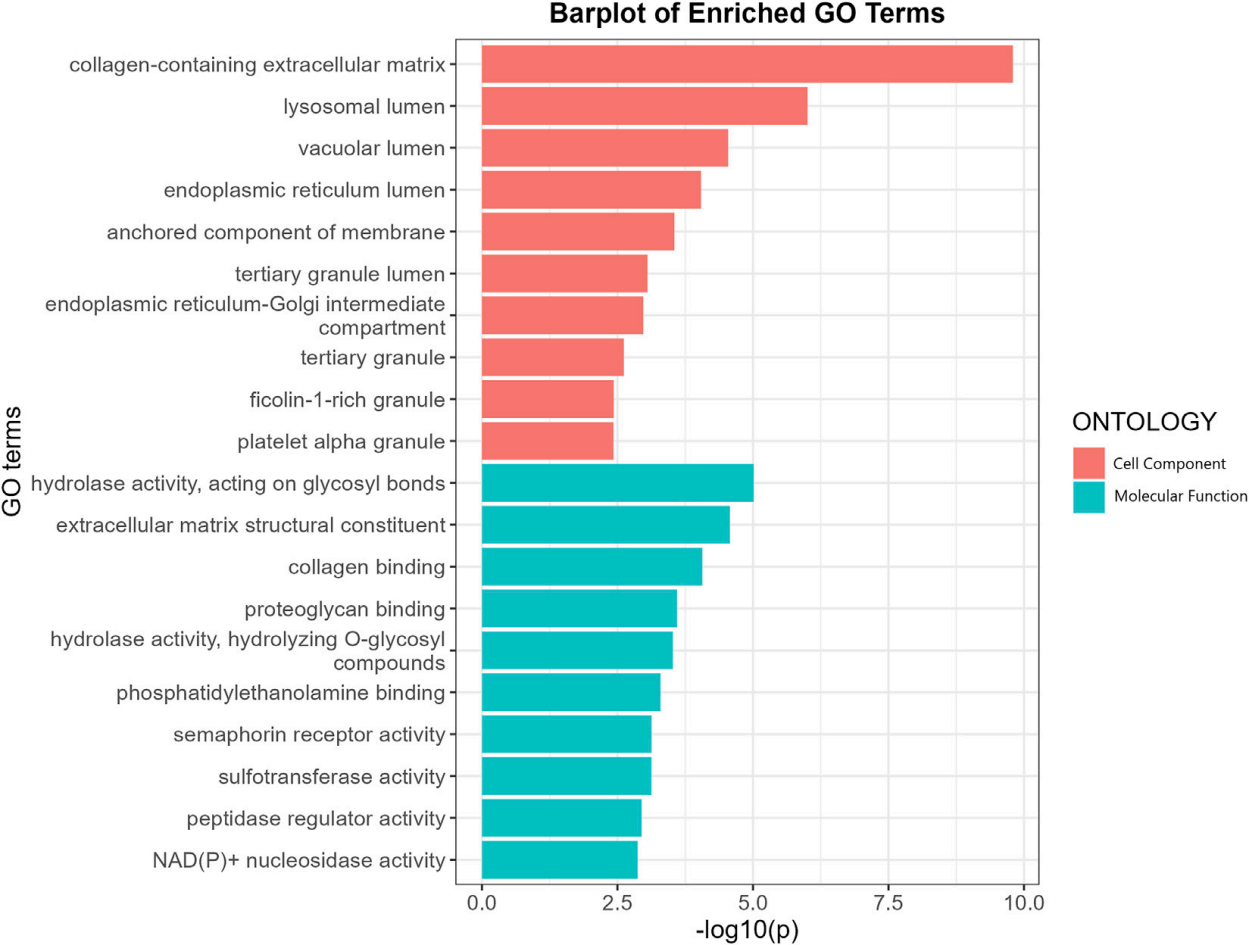
GO enrichment analysis of the putative causal proteins of BMD. *X*-axis indicates negative log10-transformed *p*-values, y a-axis indicates GO items. CC, Cellular component; MF, Molecular function.

GO enrichment analysis revealed that the putative causal proteins were involved in 37 GO items, including 12 items in the group of cellular components items and 25 items in the group of molecular function (Figure 4; Supplementary Table S5). The top GO item was collagen-containing extracellular matrix (GO:0062023, *p* = 1.6 × 10^−10^, *p*.adjust = 2.1 × 10^−8^) with 14 genes involved, including CTSB, MGP, FN1, MBL2, GDF15, OGN, ASPN, HSP90B1, SMOC2, MFAP2, SERPING1, ANXA11, DPT, CTSS. Other items, such as collagen binding (GO:0005518, *p* = 8.6 × 10^−5^, *p*.adjust = 0.006), extracellular matrix structural constituent (GO:0005201, *p* = 2.7 × 10^−5^, *p*.adjust = 0.003) has also been highlighted. We have also performed another GO enrichment analysis of non-significant proteins, while collagen binding extracellular matrix structural constituent were not among the top GO terms. We conducted an additional Gene Ontology (GO) enrichment analysis on proteins that did not show statistical significance. Our results (Supplementary Figure S1; Supplementary Table S6) did not identify collagen binding or extracellular matrix structural constituent as significant GO terms in the top-ranking results. Moreover, Many of the top enriched Gene Ontology (GO) terms for these non-causal proteins showed specificity, such as secretory granule lumen, blood microparticle, heparin binding, and receptor ligand activity. We have noticed much lower *p*-value in the enrichment analysis for non-causal proteins which may be attributed to the substantially larger sample size (1,133 vs. 67 proteins). Protein-protein interaction networks (Supplementary Figure S2) indicate FN1 and MAPK3 were highly connected with other proteins and may play a key role in the modulation of BMD.

### Druggability of the identified proteins

As shown in Supplementary Table S7, a total of 42 genes encoding putative causal proteins of BMD were listed in the druggable genome, 9 genes in tier 1 group (CTSB, MAPK3, FN1, CHST11, CA6, GSS, CD55, CTSS, MET), 7 genes in tier 2 group and the remaining 26 genes in tier 3 group. By searching Open Target Platform, we identified multiple drugs with investigational or approved indications targeting MAPK3, FN1, CA6, CTSS, MET, RSPO3, IL1RAP and IL1RL2 (Supplementary Table S8). For example, hepatocyte growth factor receptor (HGFR), encoded by MET gene, was negatively associated with BMD (beta = −0.04, *p* = 3.9 × 10^−5^), Cabozantinib may be repurposed to treat osteoporosis as an inhibitor of HGFR which was supported by real-world observation (Pan et al., 2020; Ratta et al., 2021).

## Discussion

In this study, we undertook two-sample MR analysis to investigate the causal effect of 1,245 circulating proteins from up to date largest proteomics GWAS (35,559 individuals) on multiple osteoporosis-related traits. We identified 67 proteins that were causally associated with eBMD and 38 out of the 67 proteins have been successfully replicated in other osteoporosis-related traits. The findings were supported by multiple sensitivity analysis. Further Enrichment analysis highlights the role of proteins involved in collagen binding and extracellular matrix in the development of osteoporosis. Evaluation of druggable genomics provided further information for the chance of drug repurposing to treat osteoporosis or monitoring and preventing side effects of drugs that may induce osteoporosis.

Multiple proteins that showed strong evidence to be associated with osteoporosis-related traits were supported by previous studies, such as RSPO3, IDUA, SMOC2. RSPO3 (R-spondin 3) is a secreted protein that modulates WNT-signaling pathway, and the pathway has been widely recognized to be the major determinant of bone formation and accumulation of bone mass (Marini et al., 2022). Previous GWAS study has identified locus in RSPO3 region was associated with BMD (Duncan et al., 2011). RSPO3 was highly expressed in osteoblast, and osteoblasts-specific inactivation of RSPO3 in mice induced decreased trabecular bone mass via downregulation of WNT-signaling targets (Nilsson et al., 2021). The role of RSPO3 as positive regulator of BMD was confirmed by our large-scale MR study. We have also observed a negative effect of IDUA (alpha-L-iduronidase) on BMD which is consistent with several animal studies which showed that IDUA knockout mice have thickened abnormally formed bones and increased BMD (Clarke et al., 1997; Kim et al., 2015). SMOC2 (SPARC related modular calcium binding 2 protein) is an extracellular matrix glycoprotein which promotes matrix assembly and angiogenic activity (Rocnik et al., 2006). In humans, pathogenic mutations in SMOC2 have been implicated in severe dental anomalies and skeletal dysplasia, characterized by microdontia, dentin dysplasia, reduced alveolar/jaw bone density, and flatten lumbar vertebrae (Bloch-Zupan et al., 2011; Alfawaz et al., 2013). Zebrafish models with smoc2 knockdown showed reduced expression of bone morphogenetic protein (BMP) target genes and exhibited craniofacial hypoplasia (Melvin et al., 2013; Mommaerts et al., 2014). Morkmued et al. (2020) demonstrated that deletion of SMOC2 in mouse lead to impaired bone healing and age-dependent bone loss via osteoclast activation. Our MR study further strengthens the positive effect of SMOC2 on multiple osteoporosis-related traits in the general population.

Genetically predicted higher concentration of circulating LRP4 (Low-density lipoprotein receptor-related protein 4) was positively associated with higher eBMD, TBBMD, FNBMD, FNBMD and negatively associated with lower risks of fracture in our study. However, the result was contrary to some previous studies. LRP4, belonging to lipoprotein-related protein family, is a membrane receptor. Leupin et al. (2011) found that LRP4 can interact with Sclerostin (SOST) and enhance the sclerostin-mediated inhibition of Wnt/β-Catenin Signaling and vitro mineralization. They have also identified two missense mutations (R1170W and W1186S) in sclerosteosis patients (Leupin et al., 2011). Knock-in of the mutations in mice recapitulated the high BMD phenotype (Bullock et al., 2019). Chang et al. (2014) generated osteoblast/osteocyte-specific LRP4 conditional knockout mice by flanking Lrp4 exon 1, and the mice exhibited significantly increased total bone mineral density. Here are some possible reasons for the discrepancy between our findings and these studies. Intracellular and extracellular LRP4 may exert opposite effects on BMD, plasma LRP4 may bind to the circulating SOST and attenuate the inhibition of Wnt Signaling induced by the interaction between SOST and LRP4 in the cytomembrane. Mutations in different domainss of LRP4 may also have different effects on BMD, this is supported by another study (Choi et al., 2009) which revealed that Lrp4 deficient mutant mice, generated by introducing a stop codon into the exon 36 of LRP4, exhibited shortened total femur length and reduced BMD. Furthermore, the present MR study was based on the information on plasma LRP4 levels from the general population and the condition is distinct from loss-of-function mutations in LRP4 among specific patients. Therefore, the comprehensive and exact roles of LRP4 in bone metabolism remains to be explored.

For mendelian randomization, replication in other independent datasets is important. Storm et al. (2021) successfully replicated 15 of 31 putative drug targets for Parkinson’s disease and Chen et al. (2022) replicated 4 of 11 causal genes for aortic aneurysms. In this study, 38 out of 67 proteins were successfully replicated in other osteoporosis-related traits, supporting their important role in the regulation of bone metabolism. Low statistical power in the validation cohort, difference between methods of measurement and site-specific effect on BMD may be the reasons why other proteins were not replicated. Thus, the potential causal effect of those proteins cannot be excluded. One example is CADM1 (Cell Adhesion Molecule 1) which showed positive effect on eBMD (*β* = 0.17, *p* = 1.74E-17) in the primary analysis but failed to be replicated. CADM1 is a ubiquitously expressed gene involved in many biological processes, including cancer and spermatogenesis, and revealed as an osteoblast-specific marker for osteosarcoma (Inoue et al., 2013). Using a co-expression network for mineralizing osteoblasts, Sabik et al. (2020) identified CADM1 as the core gene modulating BMD. CADM1 has also been shown to inhibit osteoclastic bone resorption under the regulation of RANKL and NFATc1, and decreased bone mass was exhibited in Cadm1-deficient mice (Nakamura et al., 2017). These studies supported CADM1 as a protective factor for osteoporosis.

GO enrichment analysis of the putative causal proteins of osteoporosis highlighted the role of collagen and extracellular matrix in the regulation of BMD. Collagen is the primary structural components of bone which enables adhesiveness of cells and assembly of the extracellular matrix. Mutations in the multiple collagen encoding genes, like COL1A1, COL1A2, COL2A1, have been well documented in a range of mendelian bone fragility disorders characterized by low bone mass and microarchitectural deterioration of bone tissue, including osteogenesis imperfecta, Ehlers–Danlos syndrome and hypochondrogenesis. Randomized controlled trials have shown that specific collagen peptides have anabolic influence on bone formation and BMD in Postmenopausal Women. Extracellular matrix (ECM) is non-cellular structure secreted by cells into the extracellular space, and has been revealed to influence functional characteristics of the mature bone by regulating cell adhesion, proliferation, and responses to cytokines. As an essential glycoprotein of the extracellular matrix, FN1 (Fibronectin 1) was highlighted as a key causal proteins in the PPI network analysis. Therefore, our result reinforced and expanded the effect of collagen and ECM on BMD among general population.

We have observed a recently published MR study (Han et al., 2023) which also investigated the potential causal effect of plasma proteins on bone mineral density. However, it is essential to emphasize that our study significantly diverges from that particular study in terms of methodology, results, and conclusions. Specifically, our study differs notably in the selection of instrumental variables for exposures. While that similar study selected all SNPs with *p* ≤ 5 × 10^−8^ across all the genomic region, we only selected cis-pQTLs, which are variants located near or within the corresponding protein-coding gene. Cis-pQTLs have higher biological plausibility, are more likely to directly affect the genes, and are less likely to be prone to horizontal pleiotropy compared to trans-pQTLs. In addition, we only included proteins with more than 3 instrumental variables, which allowed for sensitivity analyses. Our different selection criteria resulted in distinct results from the study by Han et al. (2023). For instance, in their study, they presented contradictory results with no proper explanation that elevated genetically predicted abundance of LRP4 was associated with higher eBMD and lower TB-BMD. In contrast, in our study, with the only inclusion of cis-pQTLs, elevated genetically predicted abundance of LRP4 was consistently associated with higher eBMD, TB-BMD, FN-BMD, LS-BMD, and lower risks of fracture. Moreover, we adopted the Bonferroni correction for multiple tests to minimize false positive rates, which is stricter than the false-discovery rate (FDR) correction method used by Han et al. Additionally, we used multiple datasets to replicate our primary results, including GWAS for TB-BMD, FN-BMD, LS-BMD, and fractures. These measures lowered the number of identified proteins and made our results more conclusive and credible. To further support our findings and enable their application in clinical practice, we investigated the druggability of the identified proteins, which was not included in the study by Han et al. (2023). Interestingly, some of our findings have been supported by previous observations, which further strengthen the credibility and reliability of our results. For example, hepatocyte growth factor receptor (HGFR), encoded by the MET gene, was negatively associated with BMD (beta = −0.04, *p* = 3.9 × 10^−5^). Cabozantinib, an inhibitor of HGFR, may be repurposed to treat osteoporosis, as supported by real-world observations.

Overall, our study has some strengths. MR approach can largely reduce the measured or unmeasured confounding and reverse causation compared with traditional observational studies. Therefore, the identified proteins are more like to be the “true” causal factors rather than bystanders. The large sample size of the GWAS datasets used in the study enable us to select multiple IVs for each protein which increased statistical power and reduce chance findings. Only cis SNPs which has high biological plausibility were selected as IVs. Hence, the risks of pleiotropy were minimized which strength MR results.

There are also several limitations in the present study need to be acknowledged. Firstly, the potential influence of directional horizontal pleiotropy could not be completely excluded, although we select IVs using stringent criteria and performed multiple sensitivity analyses which accounted for the horizontal pleiotropy. Secondly, the GWAS data utilized in the study were mainly obtained from the cohorts of European ancestry. Considering the difference of genetic architecture among different ethnic groups, caution should be exercised when generalizing our results to other ancestries. Further population-specific MR studies were warranted to cross-validate our findings in non-Europeans. Thirdly, we used GWAS summary data on eBMD rather than BMD in the primary MR analysis because of the larger sample size, which may induce bias. However, there is a high degree of genetic concordance between eBMD and DXA derived-BMD and we replicated the primary findings in independent GWAS datasets on DXA-derived BMD at individual bone sites and overall fracture risks. Finally, to minimize the false positive rates, we adopted Bonferroni correction for multiple tests and some proteins that may play an important role in BMD may be missed.

In conclusion, our large-scale MR analysis provided evidence that genetically predicted levels of 67 circulating proteins were associated with eBMD, and 38 of the 67 proteins were validated to be associated with other osteoporosis-related traits. Proteins involved in collagen binding and extracellular matrix play important role in the pathogenesis of osteoporosis. The study broadens the causal proteins for osteoporosis which may serve as early screening biomarkers and/or druggable targets. Further studies are warranted to validate our findings.

## Data Availability

The original contributions presented in the study are included in the article/Supplementary Material, further inquiries can be directed to the corresponding author.
